# The Development of a Nutrition Screening Tool for Mental Health Settings Prone to Obesity and Cardiometabolic Complications: Study Protocol for the NutriMental Screener

**DOI:** 10.3390/ijerph182111269

**Published:** 2021-10-27

**Authors:** Scott B. Teasdale, Sabrina Moerkl, Sonja Moetteli, Annabel Mueller-Stierlin

**Affiliations:** 1 School of Psychiatry, UNSW Sydney & Mindgardens Neuroscience Network, Sydney, NSW 2052, Australia; scott.teasdale@health.nsw.gov.au; 2 Department of Psychiatry and Psychotherapeutic Medicine, Medical University of Graz, 8036 Graz, Austria; 3 Department of Psychiatry, Psychotherapy and Psychosomatics, University Hospital of Psychiatry Zurich, 8032 Zurich, Switzerland; sonja.moetteli@pukzh.ch; 4 Department of Psychiatry and Psychotherapy II, Ulm University, 89070 Ulm, Germany; annabel.mueller-stierlin@uni-ulm.de

**Keywords:** diet, eating, mental disorders, mental health, mental illness, nutrition, nutrition screening, nutritional psychiatry

## Abstract

People living with serious mental illness (SMI) experience physical health complications at disproportionate rates to people without an SMI. Unhealthy dietary intake and disordered eating behaviors are key driving factors. There is a lack of valid nutrition-risk screening tools targeted to mental health services, and typically used nutrition-risk screening tools are not suitable for mental health services. This paper details the rationale and study protocol for development and validation of the NutriMental screener, a tool for use in clinical practice to identify service users who are at risk for common nutrition issues experienced by this population group and trigger referral to a specialist clinician. The development process includes five phases. Phase I is the development of nutrition-related domains of interest from screening tools used in mental health services. Phase II involves a literature review and service-user interviews to identify additional domains. Phase III consists of international workshops with relevant clinicians and persons with SMI to gain a consensus on questions to be included in the draft tool. Phase IV involves conducting multinational feasibility and preliminary validation studies. Phase V consists of performing formal validation studies. The development of a nutrition-risk screening tool for mental health services is a necessary step to help rectify the physical-health disparities and life-expectancy gap for people with SMI.

## 1. Introduction

Nutrition screening in hospital settings is routine clinical care in order to identify “at-risk” patients and to provide intervention by specialist clinicians. People in hospital sectors other than mental health are frequently recovering from injury or an illness that increases protein and calorie needs, while potentially also having a reduced appetite, leading to high rates of malnutrition [1]. Subsequent nutrition intervention frequently involves providing additional nutrition to prevent or manage malnutrition.

The needs and priorities of people living with serious mental illness (SMI), such as major depressive disorder, bipolar disorder, and schizophrenia and related psychoses, who utilize mental health services, are considerably different to people engaged with other health services. In contrast to other hospital sectors, people engaged with mental health services are not recovering from an illness that increases protein and energy requirements, with preliminary evidence suggesting lower requirements via a lower metabolic rate [2]. In addition, treatment commonly consists of psychotropic medication—particularly antipsychotic and mood stabilizer medication—which increase appetite [3] and the risk of disordered eating behaviors, such as binge eating [4]. Apart from medication, there are indications of complex interrelations between mental illness, and disordered-eating behaviors and eating disorders [5,6]. Moreover, unhealthy eating styles, such as emotional eating and external eating, have been linked with mental-health states [7,8]. 

A systematic review and meta-analysis published in 2019 explored the dietary intake of people with bipolar disorder and schizophrenia and related psychoses [9]. This review found a higher intake of calories and sodium compared to people without mental illness. In addition, this review found that diet quality often did not meet guidelines from peak nutrition bodies and was frequently lower than people without mental illness or the general population. Diets of people living with SMI were often characterized by higher intakes of calorie-dense, nutrient-poor foods, and lower intakes of fruits, vegetables and/or fish [9]. 

These dietary habits are related to, and are likely to intensify, medication side effects, with a mean individual weight gain of 4.4 kg (aripiprazole) to 8.5 kg (olanzapine) over the first 12 weeks of treatment [10], increasing to a mean 19 kg after four years of treatment [11]. The weight gain appears to continue long-term, with a large cohort study demonstrating that weight gain continued throughout the 20-year observational period [12]. In addition, antipsychotic medications often induce metabolic abnormalities, such as increased levels of blood glucose and lipids [13]. 

These features and other frequent issues, such as high rates of sedentary behavior [14], high rates of smoking [15] and substance use [16], have driven stark health disparities of people living with SMI. Compared to people without a mental illness, people with an SMI are at a higher risk for developing abdominal obesity (odds ratio (OR) 4.43), hypertriglyceridemia (OR 2.73), metabolic syndrome (OR 2.35), low levels of high-density lipoprotein (HDL) (OR 2.35), diabetes (OR 1.99) and hypertension (OR 1.36) [17]. Physical health comorbidities are the leading cause for the 10-to-16-year reduced life-expectancy compared to those without a mental illness [18]. 

Somatic healthcare is often neglected in mental health settings, with physical comorbidities remaining undetected for longer periods of time in patients with mental illness as compared to persons without mental illness [19]. One potential reason is diagnostic overshadowing, “a process by which physical symptoms are misattributed to mental illness” [20]. In addition, psychiatrists and mental-health teams are insufficiently aware of the somatic comorbidities and metabolic monitoring procedures for their patients with psychiatric disorders [21,22], and they have little to no training in nutrition [22]. This leads to a lower use of general preventive-care services and of routine specialized somatic care in people with an SMI, as compared to the general population [23].

One method to reduce this gap is the targeted referral of “at-risk” service users to special clinicians, e.g., referral to dietitians when a service user is deemed at risk for over- or undernutrition. A recent scoping review of nutrition screening tools used in mental-health settings found a dearth of targeted and adequately validated tools [24]. The Approaches to Schizophrenia Communication—Self-Report (ASC-SR) Checklist was the only nutrition-risk screening method for overnutrition and undernutrition identified, but this is focusing on side effects of antipsychotic treatment only [25]. Another promising screening tool, the St. Andrew’s Nutrition Screening Instrument (SANSI), was developed specifically for use in an inpatient secure psychiatric setting and has undergone limited reliability and validity testing [26]. 

This paper discusses the protocol for the development and preliminary validation of a nutrition and eating-behavior risk-screening tool for mental health services in general. This targeted nutrition-screening tool will identify mental-health-service users with possible nutrition risks, including both overnutrition and undernutrition, requiring referral to and assessment from a specialist clinician, such as a dietitian.

## 2. Materials and Methods 

**Figure 1:**
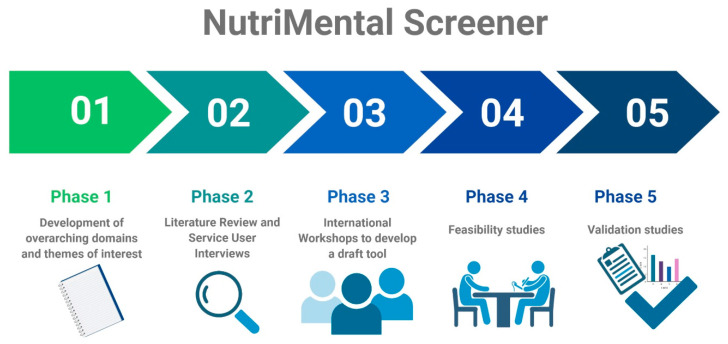
Development and validation phases of the NutriMental Screener tool. This figure was created with biorender.com (accessed on 7 October 2021).

The development and validation of the nutrition and eating-behavior risk-screening tool, called the NutriMental Screener, for people living with SMI will occur over five phases (Figure 1). Phase I and part of Phase II (“service-user interviews”) have already been completed. As the aim of this manuscript is to outline the study protocol, the report of the already obtained results is outside the scope of this manuscript. Results of all phases will be published subsequently.

### 2.1. Phase I: Development of Overarching Domains and Themes of Interest

In Phase I, complete versions of the 17 tools identified in the recent scoping review by Hancox et al. (2021) were obtained, and individual questions/items were extracted and collated [24]. Each of the 194 questions/items was inductively coded in a consensual procedure by authors AMS and ST. The resulting six overarching domains and 26 themes are presented in Table 1. 

### 2.2. Phase II: Literature Review and Service User Interviews

In Phase II, published literature is being reviewed to understand key issues and challenges faced by people with SMI related to food and eating. The findings are being used to fill gaps in the domains under consideration. Two reviews aimed to understand the dietary intake of people with bipolar disorder and schizophrenia and related psychoses [9] and with major depressive disorder [26]. Another review aims to understand the breadth and severity of disordered eating and eating behavior issues experienced by people with SMI [27]. A final review aims to understand the frequency and severity of food insecurity experienced by people with SMI [28]. 

Further, service-user interviews were conducted by the authors across three mental-health sites (Sydney, Australia; Günzburg, Germany; and Graz, Austria). The aim of the semi-structured interviews is to explore barriers and challenges to a healthy dietary pattern and impact of disordered-eating behaviors experienced by people living with SMI. Twelve participants were recruited from the site in Sydney, and eight participants each from the sites in Günzburg and Graz. Thematic analysis [29] was conducted (1) to identify additional domains and themes and (2) to gain insight into the relevance of the various themes from the participants’ perspective for discussion in Phase III of the tool development (currently unpublished). 

### 2.3. Phase III: International Workshops to Develop a Draft Tool

An international expert working group was established, comprising a range of stakeholders: dietitians, psychiatrists, clinical psychologists, nursing specialists and peer workers. The working group will engage in two online workshops and an online survey. In the first online workshop, the rationale and the overall design of the NutriMental Screening will be discussed. Moreover, the domains and themes identified in Phases I and II will be presented to, and discussed within, the working group, with consideration of preliminary findings of the further reviews and service-user interviews. The researchers aim to gain a consensus on key themes to be included in the first iteration of the screening tool. For the first draft, items from the 194-item list (Phase I) will then be selected, complemented with others if necessary, and adjusted through discourse between AMS and ST. 

An online survey will then be sent to the working group to rate the relevance of key items on a 5-point Likert scale (from “not relevant” to “very relevant”). Comments on relevance and wording of single items will be gathered by using free text fields.

In the second online workshop, findings of the online survey will be presented and discussed within the working group until consensus on inclusion and wording of each item is reached. 

### 2.4. Phase IV: Feasibility Studies

In Phase IV, the developed tool will undergo pilot testing within mental-health sites in Sydney, Australia (English language); Graz, Austria (German translation); and Zürich, Switzerland (German translation), to gain first insights in feasibility and preliminary validity. The screening tool will have an accompanying implementation manual to guide clinicians for its use.

The original NutriMental Screener will be developed in English. For feasibility testing in Austria and Switzerland, the NutriMental Screener will undergo forward–backward translation to German [30]. The German investigators (AMS, SaM and SoM) will discuss the forward translated NutriMental Screener regarding (i) spelling and grammar and (ii) local appropriateness. Finally, the local version will be backward translated into English. Both English versions will be compared, and a harmonization web conference will take place between a first-language English investigator (ST) and first-language German investigators (AMS, SaM and SoM). 

While the pilot testing of the NutriMental Screener will be embedded in a prospective observational study in Switzerland, it will be implemented in routine care in Austria and Australia. Participants will comprise people with early psychosis/at-risk mental states, schizophrenia and other related established psychoses, major depressive disorder and bipolar disorder. The researchers aim to recruit at least 60 participants living with SMI for each setting: (1) inpatients in Austria, (2) inpatients in Australia, (3) outpatients in Australia, (4) inpatients in Switzerland and (5) outpatients in Switzerland. Furthermore, about 60 adults from the general population will be recruited in Switzerland in order to compare data between people living with and without SMI. The recruitment will take place over a period of 9 months. As no inference statistics are intended to be applied, a formal sample-size estimation is not applicable. Descriptive analyses will be conducted in order to gain first insights into the feasibility and preliminary validity of the NutriMental Screener. 

A sample size (n = 60/setting) was chosen based on practical considerations as to the recruitment process (6 or 7 participants/month and setting). Nevertheless, the targeted sample size of five groups of 60 participants (300 participants) has been used in other feasibility studies for nutrition screening tools in mixed populations [31]. In Austria and Australia, the German translation and English version, respectively, will be implemented via mental-health nurses. Case-wise feasibility data that will be collected include the following: number of nurses who agree to use the tool, proportion of the nurses’ clients for which the tool is completed on, time to complete the tool, ease of use for the nurse and understanding of the questions from the service user and fidelity of the model described in the implementation manual. In addition, nurses will be asked to record whether they would have referred to a dietitian in the absence of this tool. General feasibility outcomes will be obtained bi-annually from a predesigned 32-item feedback questionnaire including general ratings, ratings on acceptability, content, delivery/practicability, usefulness of instructions and effectiveness, as well as open questions on satisfaction and suggestions for revisions. 

In Zürich, Switzerland, the German translation will be implemented via trained research associates. The feedback questionnaire on general feasibility will be applied in the mid and at the end of the 12-month recruitment phase. For preliminary validity testing, the researchers will explore associations between the nutrition-risk tool and dietary intake, eating behavior, nutritional knowledge and attitudes, and physical- and mental-health outcomes (e.g., comorbidities and psychosocial impairment).

### 2.5. Phase V: Validation Studies

Following feasibility testing, alterations will be made as required, and then formal validation studies will be conducted. Given the hybrid model of assessment that may include domains of medical history, eating behaviors and food access, multiple steps need to be taken for construct validity. This includes comparison against validated nutrition and eating-related questionnaires, testing for associations with the presence or development of cardiometabolic complications and evaluation of predictive values for health-service use. Test–retest and inter-rater reliability will be assessed. 

## 3. Discussion

**Figure 2:**
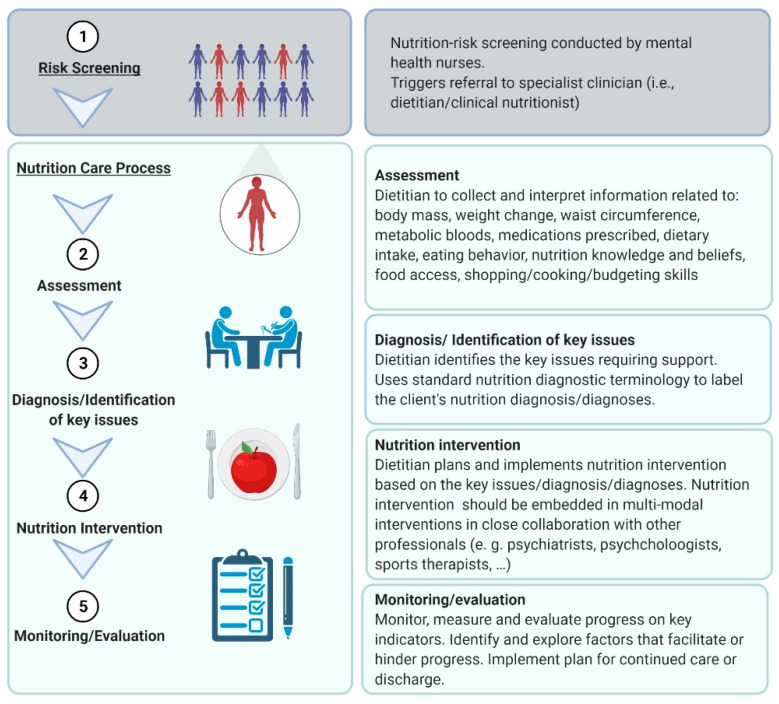
Identification and referral of patients at risk via the NutriMental Screening Tool. This figure was created with biorender.com (accessed on 7 October 2021).

There is potential for the mental-health-service-specific tool to be used as routine clinical care similar to malnutrition screening tools commonly used in other hospital sectors, such as internal medicine or surgery. Malnutrition in other hospital sectors can negatively influence patient outcomes and quality of life, and therefore early identification is employed through systematic screening on hospital admission, using simple and rapid first-line tools [32]. Patients identified as “at-risk” via the screening tool undergo a more comprehensive nutritional assessment by a specialist clinician with subsequent nutrition care plans as necessary [32]. The researchers envision that the mental-health-service-specific nutrition-risk screening tool will act in similar way, triggering referral to a specialist clinician, such as a mental-health dietitian for comprehensive assessment and development of a nutrition care plan (Figure 2).

Once a suitable tool is available for routine use in mental health services, the researchers anticipate implementation studies and quality improvement projects, and staff upskilling will be required. Mandated malnutrition screening in mental health services appears inconsistent, and rates of completion in services with mandated malnutrition screening is unknown. Lessons can be learned from the introduction of mandatory metabolic monitoring in mental health services which was met with concerningly low rates of adherence [33], and interventions designed to improve adherence were required [34]. Successful strategies can inform methods to increase adherence for nutrition-risk screening. 

The role of dietitians and clinical nutritionists in mental health services has been clearly defined [35], and the dietetic and clinical nutritionist workforce in mental health services is growing, but the frequency of these specialist clinicians working in mental health services remains sporadic, and the frequency and quality of intervention being provided is inconsistent. Mental health systems need to continue to work towards building multidisciplinary teams that include dietitians or clinical nutritionists with mental-health expertise. 

The development process has highlighted a further gap requiring investigation. To date, no dietary assessment tool or method has been validated specifically in people with SMI [36,37,38]. Given the symptomology and cognitive barriers frequently experienced by people with SMI, it cannot be assumed that currently available tools and methods are accurate and reliable. This affects both the rapidly growing field of nutritional psychiatry research and routine clinical practice, as dietitian or clinical nutritionist assessment generally includes dietary-intake assessment. 

Strengths with the development process include the following: (i) a comprehensive approach to identifying potential themes of interest; (ii) an international and multidisciplinary approach; (iii) the inclusion of people with lived experience of a SMI in the development process (qualitative interviews and workshops), also to be included in feasibility and validity studies; (iv) the use of a consensus process to develop questions to be included in the draft tool for feasibility testing; and (v) multinational pilot feasibility and preliminary validation studies before final validity testing occurs. Limitations include (i) lived experience of SMI is consultative rather than an official co-design or co-development process, and (ii) not all analyses in Phase II were fully completed before Phase III began, and thus Phase III is based on preliminary Phase II findings.

## 4. Conclusions

The development and validation of a nutrition and eating-behavior risk-screening tool specific to mental health services is critical and a positive step for rectifying the premature mortality associated with nutrition-related physical illness. If found to be valid and reliable, the NutriMental screening tool could be implemented as routine assessment in mental health services. 

## Figures and Tables

**Table 1:** Domains and themes development.

Domain	Themes
Risk factors related to disordered eating behavior	food preoccupation little appetite (undernutrition) much appetite (overnutrition) speed of eating loss of control eating food craving night eating eating structure food preferences
Risk factors related to eating behavior and connected emotions	eating shame/guilt emotional eating eating for a positive outcome
Risk factors related to body weight/shape and connected emotions	weight change thinness weight preoccupation body dissatisfaction
Risk factors related to general health state	memory/concentration swallow ability dry mouth/hypersalivation bowel habits other bodily issues
Risk factors related to the treatment of mental disorder	medication
Risk factors related to lifestyle	dieting compensatory behaviors exercise sleep hygiene
